# Three Complete Genome Sequences of Genotype G Mumps Virus from the 2016 Outbreak in Arkansas, USA

**DOI:** 10.1128/genomeA.00555-17

**Published:** 2017-08-10

**Authors:** Duah Alkam, Thidathip Wongsurawat, Piroon Jenjaroenpun, Skylar Connor, Charlotte Hobbs, Trudy M. Wassenaar, Se-Ran Jun, Intawat Nookaew, David Ussery

**Affiliations:** a Department of Biomedical Informatics, College of Medicine, The University of Arkansas for Medical Sciences, Little Rock, Arkansas, USA; b Department of Pediatrics, College of Medicine, The University of Arkansas for Medical Sciences, Little Rock, Arkansas, USA; c Molecular Microbiology and Genomics Consultants, Zotzenheim, Germany; d Department of Physiology and Biophysics, College of Medicine, The University of Arkansas for Medical Sciences, Little Rock, Arkansas, USA

## Abstract

We present here the complete genome sequences of three mumps virus (MuV) strains isolated from patients who tested positive for the mumps virus during a mumps outbreak in Springdale, AR (USA), in 2016. The virus genomes, sequenced with Oxford Nanopore technology, belong to genotype G and have an average length of 15,342 nucleotides (nt).

## GENOME ANNOUNCEMENT

Mumps is a highly contagious viral disease which is contracted through exposure to infected respiratory secretions and saliva. Patients typically present with parotitis and orchitis, while sequelae, such as meningitis and encephalitis, are not uncommon. Mumps virus (MuV), a nonsegmented, single-stranded, negative-sense RNA virus, is a member of the *Paramyxoviridae* family and belongs to the genus *Rubulavirus*. It is hypothesized that MuV infects the respiratory epithelia where it replicates and then spreads to the lymph nodes, which in turn causes viremia, enabling the virus to spread to bodily fluids (cerebrospinal fluids, urine, and serum) (1, 2).

In August 2016, a mumps outbreak began in northwest Arkansas, eventually resulting in about 3,000 infections. As of 13 April 2017, the Arkansas Department of Health reported 2,930 mumps cases. The majority of infected individuals were school-aged children who had received the measles, mumps, and rubella (MMR) vaccination (http://www.healthy.arkansas.gov/programsServices/infectiousDisease/CommunicableDisease/Pages/Mumps.aspx). We report here the complete genome sequences of three strains of MuV generated on Oxford Nanopore: Springdale_730, Springdale_745, and Springdale_754. All strains were isolated from saliva (obtained by buccal swabs) of patients who tested positive for MuV by reverse transcription-PCR (RT-PCR) of the small hydrophobic (SH) gene during the 2016 Arkansas outbreak (3).

MuV RNA was isolated from all three strains using the ZR Viral RNA Kit (Zymo Research), according to the manufacturer’s instructions. Approximately 5 ng of MuV RNA was used as the template in 4 RT-PCR reactions for strain Springdale_754 or 5 RT-PCR reactions (strains Springdale_730 and Springdale_745) using the SuperScript III one-step RT-PCR system with Platinum *Taq* high-fidelity DNA polymerase (Invitrogen). The five-reaction protocol was performed with 10 primers, provided by Biao He (4), while the four-reaction protocol used a set of 8 custom-designed primers. Amplicons were purified using DNA Clean & Concentrator-5 (Zymo Research).

For each sample, approximately 0.2 pmol each amplicon was combined and subjected to DNA sequencing library preparation using the SQK-LSK108 kit (Oxford Nanopore Technologies). All strains were sequenced on FLO-MN106 flow cells mounted on an Mk1b MinION device. Data acquisition was performed using MinKNOW software version 1.5 to generate the read files. The raw signal from the read files was used to call corresponding bases by Albacore version 0.8.4 using standard configuration for the SQK-LSK108 kit. After base calling had been carried out, the reads, which have a mean quality score of >9, were kept for the next step. Consensus sequences of 15,351 bp (average depth, 2,263×), 15,338 bp (average depth, 2,388×), and 15,338 bp (average depth, 2,267×) for Springdale_754, Springdale_730, and Springdale_745, respectively, were generated using the modified ZiBRA analysis pipeline (http://www.zibraproject.org/data). All three strains clustered within a clade of genotype G on a maximum likelihood tree of MuV complete genomes (extracted from GenBank). The three Springdale strains shared a sequence identity of at least 99.4% among them and 99.4% with a reference genome used for template assembly (accession no. KF481689.1). We also demonstrate here the practicality of the Oxford Nanopore Technology, as we swiftly sequenced the mumps virus from an active outbreak.

### Accession number(s).

The three mumps virus genome sequences have been deposited in GenBank under accession numbers KY996512, KY996510, and KY996511.
